# Notch1 haploinsufficiency in mice accelerates adipogenesis

**DOI:** 10.1038/s41598-021-96017-z

**Published:** 2021-08-18

**Authors:** Kazutoshi Yamaguchi, Motoharu Hayashi, Yasuhiro Uchida, Xian Wu Cheng, Takayuki Nakayama, Tadashi Matsushita, Toyoaki Murohara, Kyosuke Takeshita

**Affiliations:** 1 Department of Cardiology, Nagoya University Graduate School of Medicine, Nagoya, Japan; 2 Department of Cardiology/Hypertension and Heart Center, Yanbian University Hospital, Yanji, Jilin China; 3 Department of Community Health and Geriatrics, Nagoya University Graduate School of Medicine, Nagoya, Japan; 4 Department of Blood Transfusion, Aichi Medical University Hospital, Nagakute, Japan; 5 Department of Clinical Laboratory, Nagoya University Hospital, Nagoya, Japan; 6 Department of Blood Transfusion, Nagoya University Hospital, Nagoya, Japan; 7 Department of Clinical Laboratory, Saitama Medical Centre, Saitama Medical University, 1981 Kamoda, Kawagoe, Saitama Japan

**Keywords:** Obesity, Experimental models of disease, Cell signalling

## Abstract

Notch signaling has been recognized recently as a key regulator of metabolism. Here, we determined the role of Notch1 in adipogenesis in wild-type (WT) and Notch1 hetero-mutant (N1+/−) mice provided with 12-week normal or high-fat diet. Haploinsufficiency of Notch1 was associated with adipose tissue accumulation despite similar food intake. White adipose tissue (WAT) of N1+/− showed accumulation of adipogenic cells (CD34+CD68+ cells), crown-like structures, and upregulation of cell proliferation markers (cyclin D1 and Ki67). Notch1 expression in WAT reached peak levels in 8-week-old WT mice in parallel with fat accumulation, especially under HF/HS-feeding, whereas such increment was blunted in N1+/− mice. Downstream of Notch1 haploinsufficiency, over-expression of adipogenic factors PPARγ and C/EBPα was noted following down-regulation of downstream transcriptional factors of Notch signaling (Hes-1, Pref-1, and Sox9). Both pharmacological Notch signal inhibition and Notch1 knockdown enhanced adipogenesis of 3T3-L1 preadipocytes. N1+/− mice showed impaired glucose and insulin tolerance with downregulation of IRS-1 and GLUT4 in WAT after high-fat diet. Taken together, our results suggest that haploinsufficiency of Notch1 promotes fat accumulation and adipogenesis and provides a mechanistic link between Notch signaling and development of metabolic syndrome.

## Introduction

Notch signaling is recognized as a highly conserved pathway involved in the regulation of various cellular functions, including proliferation, differentiation, and apoptosis^1,2^. Once activated by transmembrane ligands, Notch receptors (Notch1-4) result in proteolytic cleavage and release of Notch intra-cellular domain (NICD) into the nucleus. NICD binds with the RBP-J protein, and activates the transcription of several downstream target genes, such as hairy enhancer of split homolog-1 (Hes-1) and Hairy/enhancer-of-split related with YRPW motif protein 1 (Hey-1)^1,2^.

The Notch signaling pathway plays an active role in the entire adipogenesis process, including proliferation and differentiation of adipocyte progenitor cells and has recently been recognized as a key player in metabolism^3,4^. Differentiation of preadipocytes to mature adipocytes during adipogenesis involves a complex network of transcription factors. Among these, peroxisome proliferator-activated receptor γ (PPARγ) and CCAAT/enhancer binding protein α (C/EBPα) are recognized as key activators of adipogenesis, and cooperative interaction between the two is well established^5^. Preadipocyte factor-1 (Pref-1), which is strictly regulated by Notch signal activity, is a transcriptional factor known to inhibit adipogenesis^6^. Pref-1 is highly expressed in preadipocytes but absent after adipocyte differentiation^6^. Overexpression of Pref-1 resulted in impaired adipogenic differentiation, while increased adipogenic differentiation was observed following knockdown of Pref-1 gene^6^. SRY (sex determining region Y)-box 9 (Sox9), which is a potent target molecule of Pref-1 via MEK/ERK signal activation, binds to the promotor region of C/EBPβ and C/EBPδ to suppress their promotor activities, and prevents adipocyte differentiation^6^.

Evidence suggest that the Notch signal strictly regulates cell fate via appropriate timing of activation and dosage of its downstream transcription factors^7,8^. During development, Notch signal activity oscillates to induce neurogenesis and somatogenesis during embryogenesis, while abnormal timing of activation disrupted both these two processes^7^. Meanwhile, the wild-type (WT) and haploinsufficient Notch1 gene have the opposite effects on cell protection in response to shear stress in induced pluripotent stem cell-derived endothelial cells^8^. WT Notch1 gene activated by shear stress leads to epigenetic changes at Notch1-bound enhancers and transcriptional activation of anti-calcific gene programs that prevent osteogenesis, inflammation, and oxidative stress with epigenetic modification^8^. Haploinsufficiency of Notch1 WT Notch1 gene, which cannot mediate the proper response to shear stress, results in epigenetic dysregulation and aberrant upregulation of pro-calcific and inflammation transcriptional factors. These findings suggest that proportion of pathological and physiological phenotypes does not correlate with the extent of Notch signal suppression. Indeed, differences in experimental setting (e.g., pharmacological or genetic interference, and time course) of Notch signal suppression could alter its effects of adipogenesis^4^.

During breeding of the Notch1 heterozygous-deficient (N1+/−) mice, we incidentally found that the N1+/− mice can gain weight easily. Based on this finding, we hypothesized the involvement of Notch1 haploinsufficiency in the derepression of anti-adipogenic pathway via Pref-1. To test our hypothesis, we conducted three experiments in the present study. First, we defined the pathological findings in the adipose tissues of lean and obese N1+/− and their WT littermates, including adipose tissue accumulation, and altered expression levels of adipogenic transcriptional factors. Second, we examined adipogenesis in vitro in Notch1 signal-inhibited 3T3-L1 preadipocytes and adipose-derived stem cells (ADSCs) prepared from N1+/− and WT littermates. Third, we evaluated the metabolic changes in N1+/− to test our hypothesis that Notch1 facilitates adipogenesis and alters systemic metabolism.

## Material and methods

### Animal experiments

#### Animals and diet

Notch1 heterozygous-deficient (N1+/−) mice with C57BL/6J background (Jackson Laboratory) and wild-type (WT) mice were generated as described previously^9^. At 4 weeks of age, the male mice were housed two per cage under standard conditions (23 ± 1 °C, 50 ± 5% humidity), with a 12-h light/dark cycle in a viral pathogen-free facility and handled properly accordingly. Starting from at 4 weeks of age, mice were randomly assigned to a normal diet (n = 10) or high-fat high-sucrose (HF/HS) diet (n = 10) (F2HFHSD, Oriental Yeast CO., 54.5% kcal from fat). Body weight and food intake were monitored for 12 weeks. When either 8- or 16-week-old, the mice were sacrificed and blood, inguinal adipose tissue, and skeletal muscle samples were collected for analysis and histological examination. Plasma total cholesterol (T-cho), triglyceride (TG), and FFA levels were measured with a commercially available enzymatic kit (Wako, Japan)^10^. The animals were handled in accordance with the guidelines of the Institutional Animal Care and Use Committee of Nagoya University. The study protocol was approved by the Animal Ethics Review Committee of Nagoya University (Protocol Number 20360). All experiments performed with mice were in accordance with the ARRIVE guidelines and regulations of this committee.

#### Analysis of fat mass by magnetic resonance imaging (MRI)

Body fat composition was analyzed in both N1+/− and WT mice (n = 5, each) by MRI after 12-week-HF/HS diet (16-week-old)^11^. Briefly, each mouse was anaesthetized with isoflurane (2 vol% in air). A series of T1-weighted axial slices was obtained with MRmini SA (DS Pharma Biomedical, Osaka). The visceral adipose tissue volume (area from the diaphragm to the anus) was analyzed with software Image J (National Institutes of Health, Bethesda, MD), representing the sum of fat area in each MRI slice × slice thickness.

#### Cold exposure and body temperature measurement

WT and N1+/− mice (n = 5, each) were housed in cages at 4 °C for 8 h, and rectal temperature was recorded with a Bat-10 thermometer coupled to a RET-3 mouse rectal probe (Physitemp, Clifton, NJ) lubricated with mineral oil at a frequency of one data point per 1 h^12^. The measurement was conducted at 8 weeks of age.

#### Immunohistochemical and histological analyses

The collected adipose tissue samples were subjected to immunohistochemistry using antibodies for CD34 (dilution, 1:100, #C-18, Santa Cruz Biotechnology) and CD68 (dilution, 1:100, Santa Cruz Biotechnology)^13^, as described in detail previously^10^. Nuclei were counterstained with 4ʹ,6-diamidino-2phenylindole (DAPI, Sigma-Aldrich, St. Louis, MO). Inguinal white adipose tissue sections (5-μm thickness) were stained with hematoxylin–eosin (H&E) using standard protocols. CD 34(+) and CD68(+) double-positive cells were defined as adipogenic cell clusters^14^. CD68(+) multinuclear giant cells surrounding individual adipocytes were defined as “crown-like structure”, representing necrotic adipocytes^13^. The stained slides were analyzed in a blinded fashion by two independent investigators for the density of adipocyte size and crown-like structures under 200× magnification. The size of inguinal adipocytes was estimated using Win ROOF version 5.02 (MITANI Co, Fukui, Japan).

#### Quantitative PCR

Total RNA extraction, reverse-transcription, and quantitative PCR were performed as described previously^15^. The primer sequences used in this study are listed in Table 1. The mRNA level was normalized relative to that of β-actin mRNA.

**Table 1:** Sequences of primers used for RT-PCR.

	Forward (5ʹ–3ʹ )	Reverse (5ʹ–3ʹ)
Cyclin D1	GGATGTGAGGGAAGAGGTGA	CCCTGACTGCTGTGATGCTA
Ki67	GACAGCTTCCAAAGCTCACC	ACACTTCCTTGGGGTCCTCT
Notch1	TGTGACAGCCAGTGCAACTC	TGGCACTCTGGAAGCACTGC
Notch2	TGTGGAAGGAATGGTGGCAGAG	CTCGGGCAGCAAGAAACAAAGG
Notch3	ACACTGGGAGTTCTCTGT	GTCTGCTGGCATGGGATA
Notch4	CTGGGCAAGGAGACAGAGTC	CCACTCCATCCTCATCCACT
Hes-1	GCCAGTGTCAACACGACACCGG	TCACCTCGTTCATGCACTCG
Pref-1	TGTGACCCCCAGTATGGATT	AGGGGTACAGCTGTTGGTTG
Sox9	GAGGCCACGGAACAGACTCA	CAGCGCCTTGAAGATAGCATT
PPARγ	GTGATGGAAGACCACTCGCATT	CCATGAGGGAGTTAGAAGGTTC
C/EBPα	TAGGTTTCTGGGCTTTGTGG	GTTGGTGTTGTACGTCTTGGA
IRS-1	CCAGAGTCAAGCCTCACACA	CCCAACTCAACTCCACCACT
GLUT4	TCCCTGTTACCTCCAGGTTG	CCTTGCCCTGTCAGGTATGT
β-actin	TGGAATCCTGTGGCATCCATGAAAC	TAAAACGCAGCTCAGTAACAGTCCG

#### Western blot analysis

Adipose tissue or cell samples were homogenized and total protein concentration was measured using the Pierce BCA Protein Assay Kit (Thermo Scientific Inc., Billerica, MA). Equal amounts of proteins were separated by SDS–polyacrylamide gel electrophoresis and transferred onto polyvinylidene difluoride (PVDF) membranes (Immobilon-P, Millipore Bedford, MA). The membranes were cut according to the molecular weight and then incubated with antibodies directed against total and cleaved Notch1 (Santa Cruz Biotechnology, Santa Cruz, CA; dilution, 1:1000, Cell Signaling Technology, MA; dilation, 1:1000), Notch3 (Proteintech, Chicago, IL; dilution, 1:1000), Pref-1 (Abcam, Cambridge, UK), respectively. Then, the membranes were further incubated with HRP-linked secondary antibody (dilution, 1:2000) at room temperature for 1 h. After washing with TBS-T three times, protein expression was visualized using the enhanced Chemi-Lumi one system (Nacalai Tesque, Kyoto, Japan). The intensity of protein bands was normalized to the amount of β-actin (an internal control, Cell Signaling Technology, MA; dilution, 1:2000) and expressed as ratio (fold increase) of the control value.

#### Intraperitoneal glucose and insulin tolerance tests

At 16 weeks of age, the mice fed with HF/HS were subjected to intraperitoneal glucose tolerance test (GTT) and insulin tolerance test (ITT) using standard protocols, as described in detail previously^16^. Briefly, for GTT, the mouse was fasted overnight and then challenged with 2 g/kg d-glucose (Sigma-Aldrich), followed by serial measurements of blood glucose up to 120 min using a blood glucose level monitor (Glutest Ace, Sanwa Kagaku Kenkyusho Co, Nagoya, Japan). For ITT, the mouse was fasted for 16 h before testing. Insulin (0.75 U/kg, Actrapid Penfill, NovoNordisk) was injected intraperitoneally, and blood glucose level was measured serially.

### In vitro experiments

#### Cell culture and reagents

All culture reagents were obtained from Sigma-Aldrich. Mouse ADSCs were prepared from 8-week-old N1+/− and WT mice, as described in detail previously^16^. 3T3-L1 preadipocytes (DS Pharma Biomedical Co., Osaka) and ADSCs of passages 4 to 8 were maintained in Dulbecco’s modified Eagle medium containing 4.5 g/L d-glucose, supplemented with 10% fetal bovine serum (FBS), 100 U/ml penicillin, and 100 µg/ml streptomycin (DMEM-HG) at 37 °C in a humidified atmosphere of 5% CO_2_. Two days after confluence, cell differentiation was initiated with a mixture of 1.67 µM insulin, 0.5 mM 3-isobutyl-1-methylxanthine and 1 µM dexamethasone in fresh DMEM-HG. After 72 h, the medium was maintained with fresh DMEM-HG supplemented with 1.67 µM insulin. After 48 h, insulin was withdrawn from the culture media and adipogenetic changes were observed^16,17^. In some experiments, the cells were treated with vehicle (dimethyl sulfoxide) or γ-secretase inhibitors (LY411,575 at 100 nM and 1 µM) during differentiation, as described previously^10^.

#### Induction of adipogenesis and oil red-O staining

3T3-L1 cells were seeded onto 6-well plates and adipocyte differentiation was induced as described above^17^. The formation of lipid droplets in the differentiated adipocytes was analyzed using oil red-O staining according to the instructions provided by the manufacturer. Briefly, the cells were fixed in 4% formaldehyde solution for 10 min, washed with phosphate-buffered saline (PBS), and stained with oil red-O solution (Diagnostic BioSystems, BH Hague, Netherlands) for 6 min. To quantify lipid droplets, the average areas of red-stained droplets were measured. The progress of adipogenesis was assessed by lipid drop density (pixels per pixels).

#### SiRNA transfection of 3T3-L1 cells

3T3-L1 preadipocytes were transfected with a Thermo Fisher Scientific small interfering RNA (siRNA) against Notch1 (siRNA-1 and -2; catalog s70700 and s70698) at 20 pmol, or nonsilencing control RNA (catalog #1027280) at 20 pmol, using Lipofectamine 2000 reagent, according to the instructions supplied by the manufacturer (Invitrogen Life Technologies, Carlsbad, CA)^9^.

#### Statistical analysis

Data are expressed as mean ± SD. Differences between groups were assessed by Student's t-test. Quantitative data between different groups were analyzed by Fisher’s protected least significant differences (PLSD) test of one-way analysis of variances (ANOVA). Results were considered significant with P < 0.05.

## Results

### Animal experiments

#### Notch1 haploinsufficiency is associated with increased adiposity

**Figure 1:**
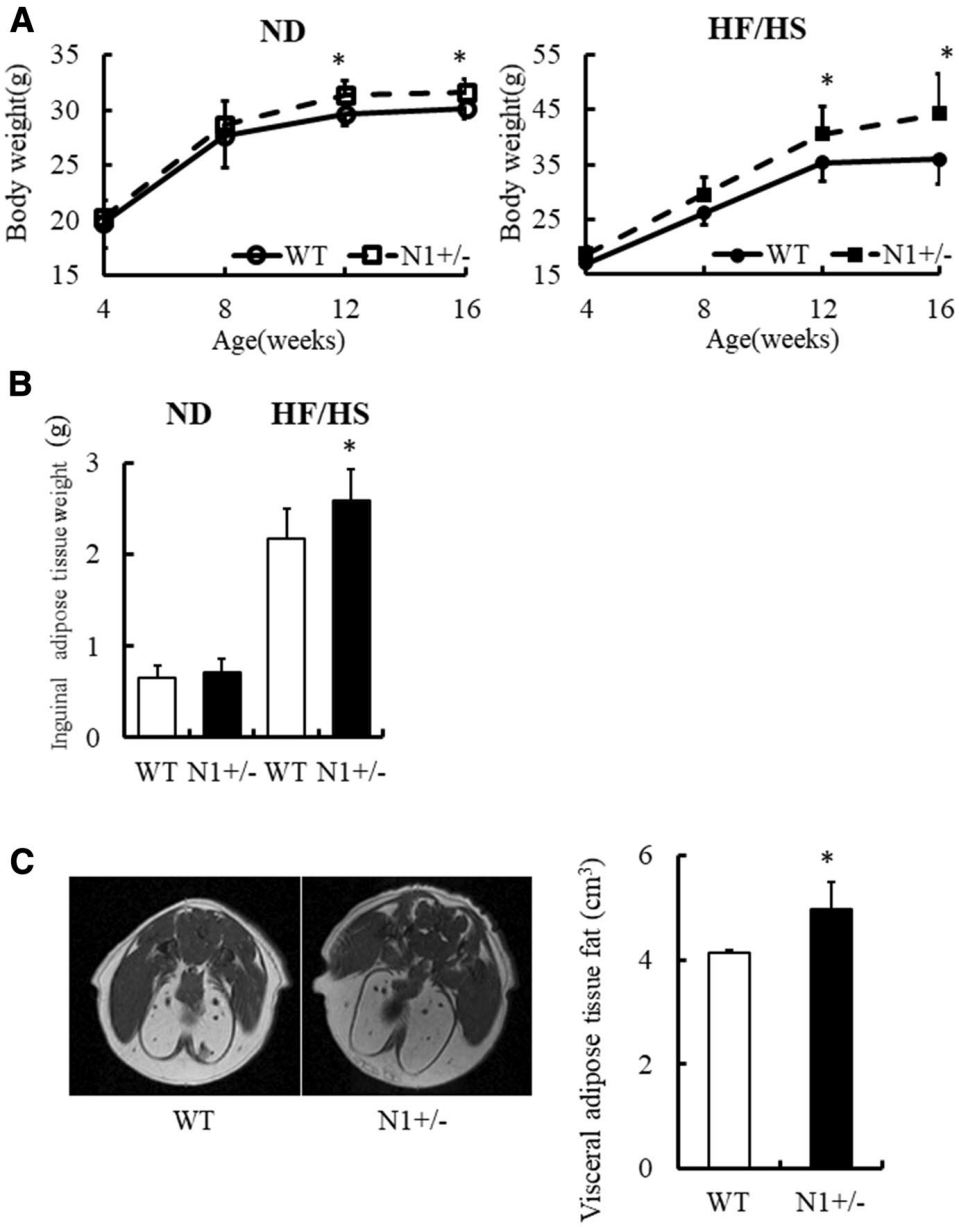
Notch1 haploinsufficiency increases adiposity. Notch1 heterozygous-deficient (N1+/−) mice gained more body weight and adipose tissue weight than wild-type (WT) mice, fed with normal (ND) or high-fat high-sucrose (HF/HS) diet. (**A**) Body weight of WT and N1+/− mice. *Left*: ND, *Right*: HF/HS. n = 10 per group. (**B**) Inguinal adipose tissue weight of 16-week-old mice. n = 10 per group. (**C**) *Left*: representative magnetic resonance imaging (MRI) of 16-week-old WT and N1+/− mice, fed with HF/HS. *Right*: Visceral adipose tissue measurements of 16-week-old WT and N1+/− mice, fed with HF/HS using MRI. n = 5 per group. Data are expressed as mean ± SD. *P < 0.05, compared with the WT mice.

To investigate whether Notch1 haploinsufficiency is associated with obesity, we fed 4-week-old male Notch1 heterozygous-deficient (N1+/−) mice and their WT counterparts high-fat high-sucrose (HF/HS) or normal diet, respectively, and measured body weight and daily food intake for 12 weeks. The increase in body weight during this period was significantly higher in the N1+/− mice compared with WT mice (Fig. 1A), although food consumption was comparable between the two groups (WT 81.4 ± 6.9 mg/g day vs. N1+/− 83.1 ± 8.6 mg/g day, P = 0.66). Body fat accumulation was further examined by measuring inguinal adipose tissue weight. The weight of inguinal adipose tissue at the end of the 12-week diet was significantly higher in the N1+/− than WT mice (Fig. 1B). This finding was supported by the presence of more visceral adipose tissue in the N1+/− mice by MRI analysis (Fig. 1C). Measurement of body temperature during cold exposure at 4 °C for 8 h showed no differences in thermogenesis between the two strains (data not shown).

#### Notch1 haploinsufficiency alters adipogenic properties

**Figure 2:**
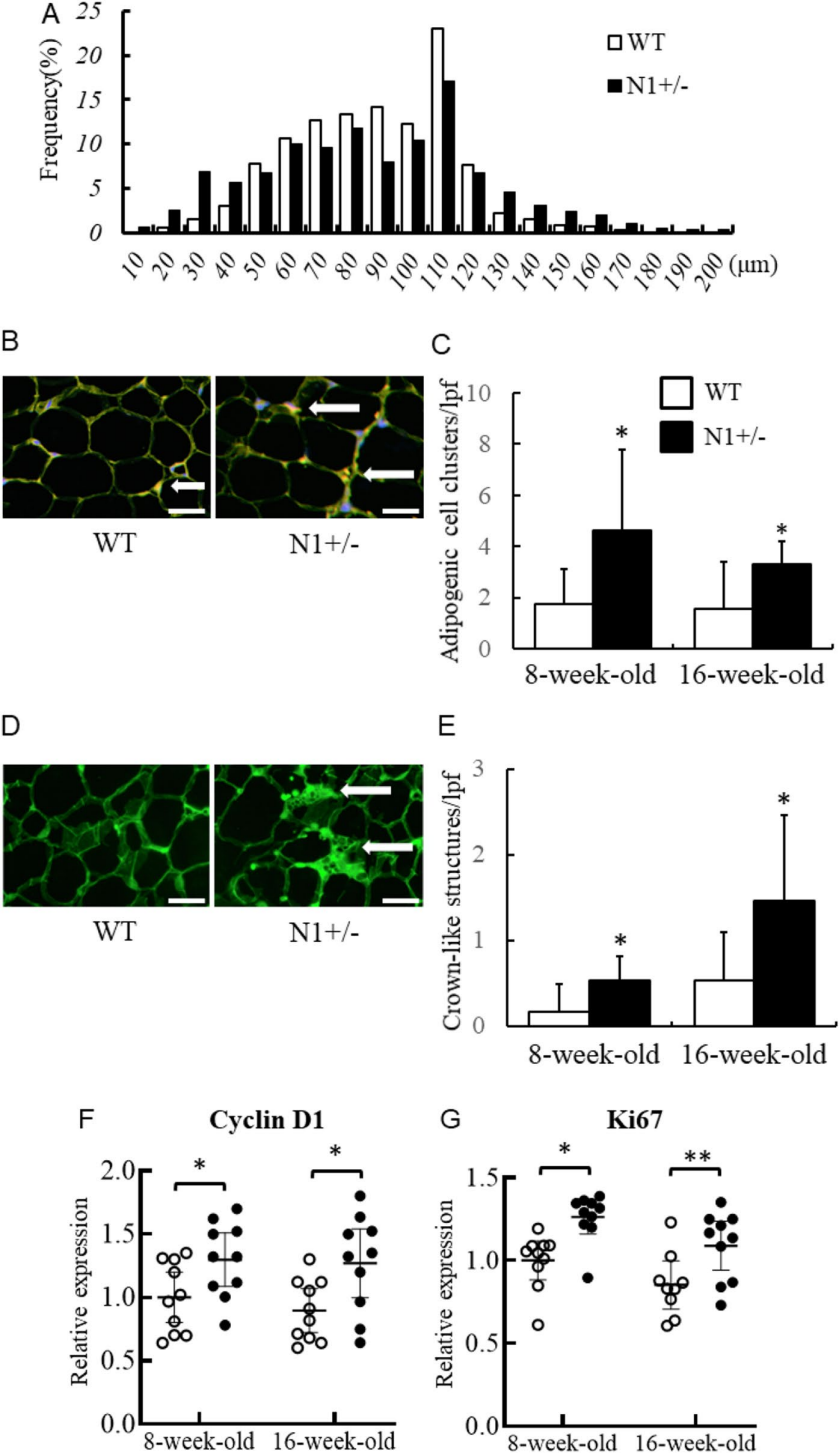
Notch1 haploinsufficiency reveals adipogenic property. Abundance of small- and large-diameter adipocytes in inguinal adipose tissue of N1+/− mice. Adipogenic cell clusters (CD34+CD68+ cells), crown-like structures (MAC-2-positive multinuclear giant cells surrounding individual adipocytes), and quantitative PCR for proliferation markers (cyclin D1 and Ki67) were increased in N1+/− mice. (**A**) Distribution of adipocyte cell diameter in inguinal adipose tissues of WT and N1+/− mice after 12-week HF/HS diet. (**B**) Immunohistochemical analysis of adipogenic cell clusters in adipose tissue of 8-week-old WT and N1+/− mice (arrows). Scale bars = 20 μm. (**C**) The percentage of adipogenic cell clusters was higher in HF/HS diet-fed N1+/− mice. (**D**) Immunohistochemical analysis of crown-like structures in adipose tissue of 8-week-old WT and N1+/− mice (arrows). Scale bars = 20 μm. (**E**) The density of crown-like structures was higher in HF/HS diet-fed N1+/− mice. (**F**) Quantitative analysis of cyclin D1 mRNA in adipose tissue of WT and N1+/− mice, fed with HF/HS diet. (**G**) Quantitative analysis of Ki67 mRNA in adipose tissue of WT and N1+/− mice, fed with HF/HS diet. Data are mean ± SD. *P < 0.05 and **P < 0.01, compared with WT mice. n = 8–10 per group.

To check the role of Notch1 haploinsufficiency in the adipogenic properties of adipocytes, we conducted morphological analysis and measured the expression levels of various proliferative transcriptional factors. After the 12-week HF/HS diet, the frequencies of small (< 40 μm) and large (> 130 μm) diameter adipocytes in inguinal adipose tissues were higher in N1+/− mice than WT mice (Fig. 2A). Furthermore, after 4-week and 12-week HF/HS diet, the number of clusters of adipocytes and crown-like structures were significantly higher in the N1+/− mice compared to the WT mice (Fig. 2B–E), where these components were not detected in both N1+/− mice and WT fed normal diet (data not shown). After HF/HS diet, the mRNA expression levels of cyclin D1 and Ki67 in the inguinal adipose tissues of N1+/− mice were significantly higher than those of WT mice (Fig. 2F,G). Those of N1+/− and WT mice were comparable after normal diet (Supplemental Figure S1).

#### Notch1 haploinsufficiency alters adipogenic transcriptional factors in white adipose tissue

**Figure 3:**
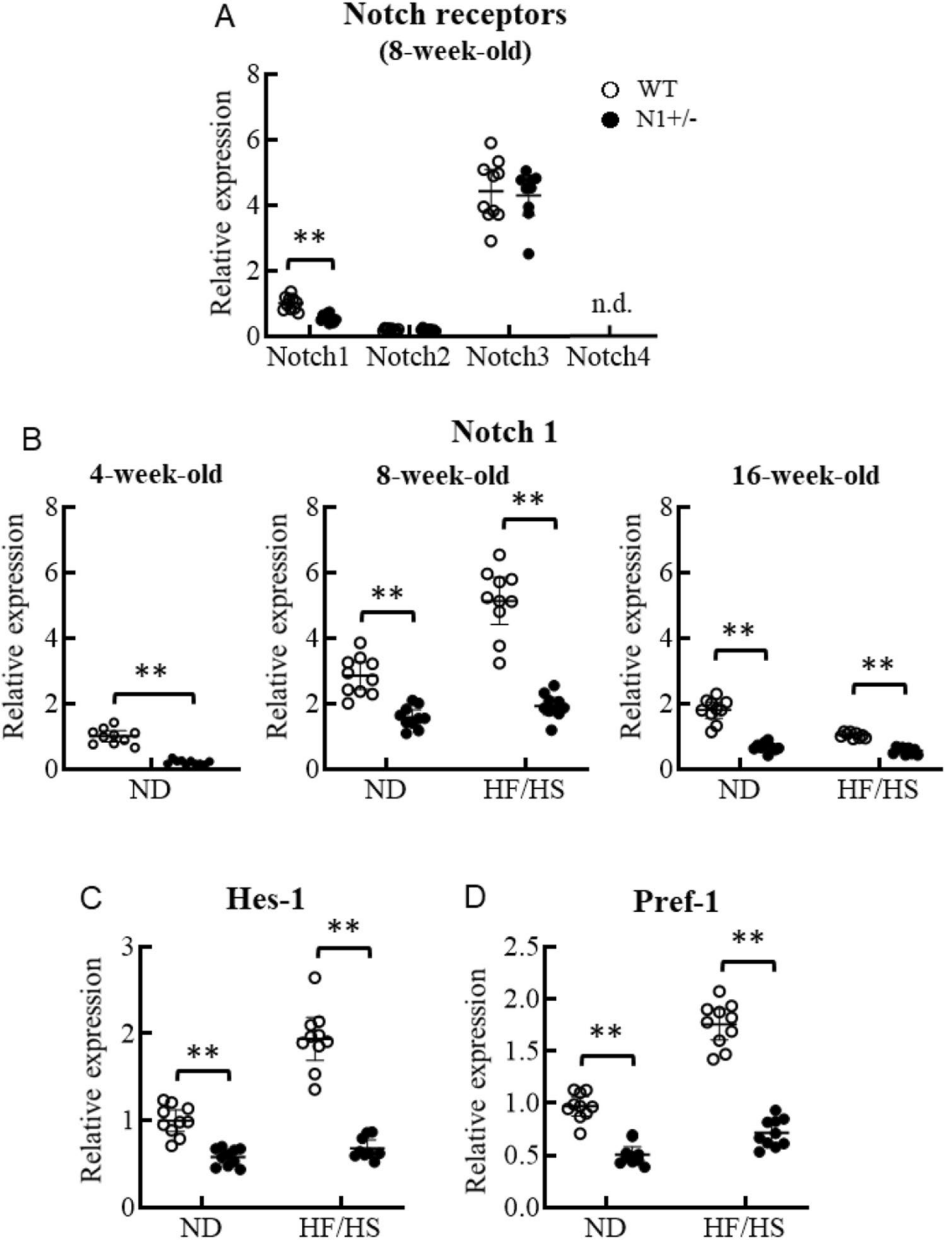

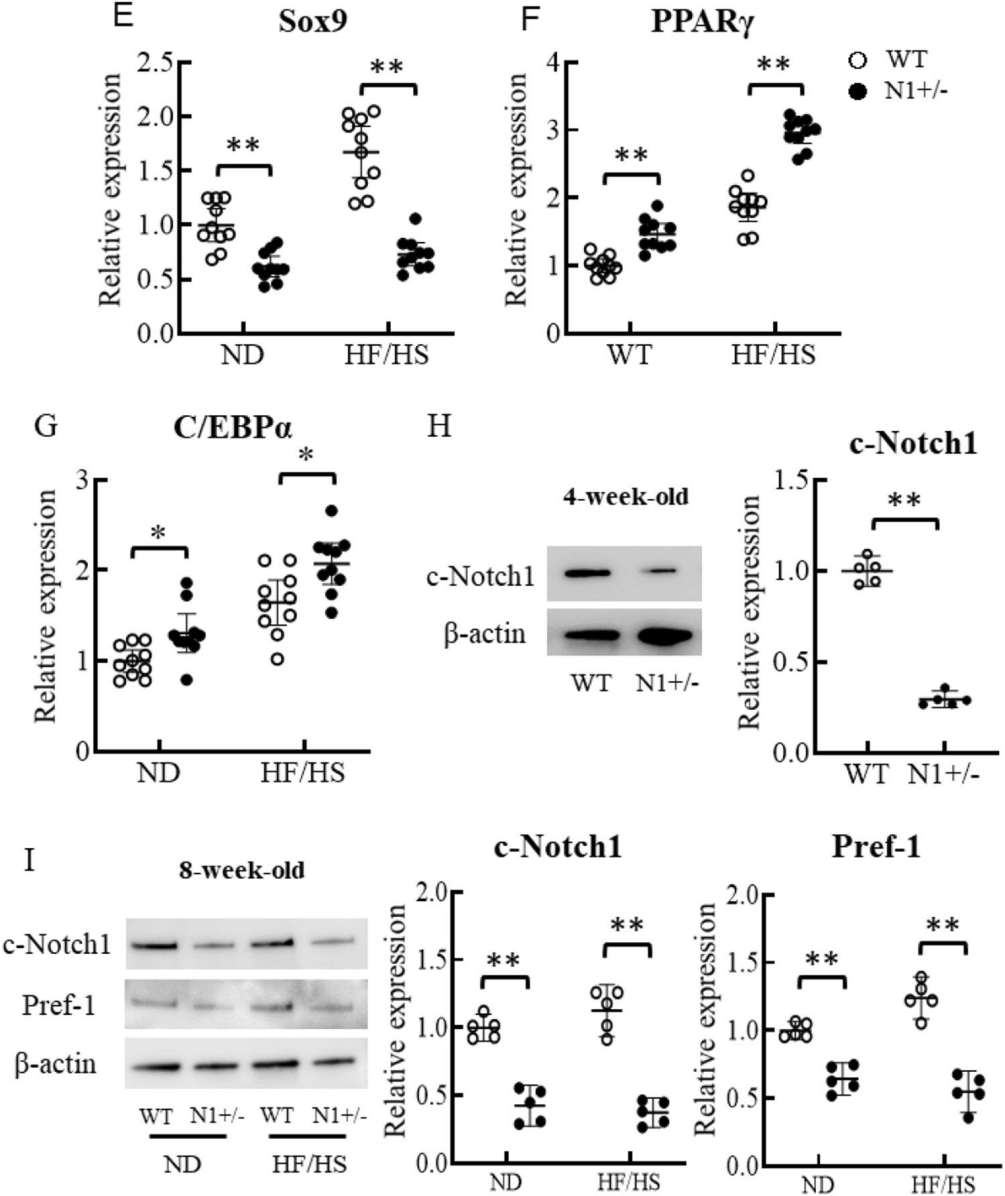
Notch1 haploinsufficiency increases adipogenic transcriptional factors in white adipose tissue. Notch1 expression in adipose tissue was decreased by as much as 50% in N1+/− mice. Notch1 expression in adipose tissue reached a peak value in 8-week-old mice in parallel with fat accumulation especially under HF/HS-fed condition, but such increase was blunted in N1+/− mice. The expression levels of Hes-1 and Pref-1, and Sox9 were decreased in adipose tissues of N1+/− mice. Downregulation of Sox9 enhanced the expression of the master activators of adipogenesis, PPARγ and C/EBPα. (**A**) Quantitative analysis of Notch1, Notch2, Notch3, and Notch4 mRNA expression levels in adipose tissues of 8-week-old WT and N1+/− mice. (**B**) Quantitative analysis of Notch1 mRNA expression levels in adipose tissues of 4-, 8-, and 16-week-old mice. (**C**–**G**) Quantitative analysis of mRNA expression levels of Hes-1 (**C**), Pref-1 (**D**), Sox9 (**E**), PPARγ (**F**), and C/EBPα (**G**) in adipose tissues of 8-week-old mice. (**H**) Immunoblotting showing cleaved-Notch1 in adipose tissues of 4-week-old mice. Representative western blot of fractionated samples. The cropped blots are used in the figure. The membranes were cut prior to exposure so that only the portion of gel containing the desired bands would be visualized. The samples of fractionation derive from the same experiment and the blots were processed in parallel. Full-length blots are shown in Supplementary information (Page 6). Quantification of western blot bands. Protein expression was normalized to β-actin. (**I**) Immunoblotting showing cleaved-Notch1 and Pref-1 in adipose tissues of 8-week-old mice. Representative western blot of fractionated samples. The cropped blots are used in the figure. The membranes were cut prior to exposure so that only the portion of gel containing the desired bands would be visualized. The samples of fractionation derive from the same experiment and the blots were processed in parallel. Full-length blots are shown in Supplementary information (Page 6). Quantification of western blot bands. Protein expression was normalized to β-actin. Data are mean ± SD. **P < 0.01, compared with WT mice. n = 8–10 per group (**A**–**G**), n = 5 per group (**H** and **I**).

At 8 weeks of age, the expression level of Notch1 in white adipose tissues of N1+/− mice was about 50% of that of WT, whereas those of other Notch homolog receptors were comparable between the two groups (Fig. 3A). In the adipose tissues of normal diet-fed WT mice, Notch1 expression level increased 2.9 ± 0.56 times at 8 weeks of age compared to that at baseline (4 weeks of age) (Fig. 3B). The peak Notch1 expression level in HF/HS diet-fed WT mice was 5.2 ± 0.96 times that recorded at baseline. On the other hand, the expression level of Notch1 in N1+/− mice was 30% lower than that of WT at 4 weeks of age, and the age-related increment in the expression was blunted up to approximately 50% of WT mice at 8- and 16 weeks of age. The expression levels of cleaved Notch1, which is constitutively active form of Notch1 receptor, were also decreased in the adipose tissues in N1+/− mice in accordance with the mRNA expression (Fig. 3H,I). We also investigated the mRNA expression of Hes-1 and Pref-1 located downstream of Notch1 signaling. Notch1 haploinsufficiency was associated with under-expression of Hes-1 and Pref-1 (Fig. 3C,D). The expression levels of Pref-1 were also decreased in the adipose tissues in N1+/− mice in accordance with the mRNA expression (Fig. 3I). The mRNA expression of Sox9, which represses PPARγ and C/EBPα to facilitate adipogenesis, was also lower, in parallel with the low levels of Pref-1 (Fig. 3E). The expression levels of PPARγ and C/EBPα, the master activator of adipogenesis, were derepressed in downstream of Notch1 haploinsufficiency (Fig. 3F,G). This tendency was especially evident in HF/HS diet-fed mice. Finally, the expression levels of markers of beiging, including UCP-1, CD137, and Tbx-1, were comparable between WT and N1+/− mice (data not shown).

#### High FFA concentration and poor glucose metabolism in Notch1+/− mice

**Figure 4:**
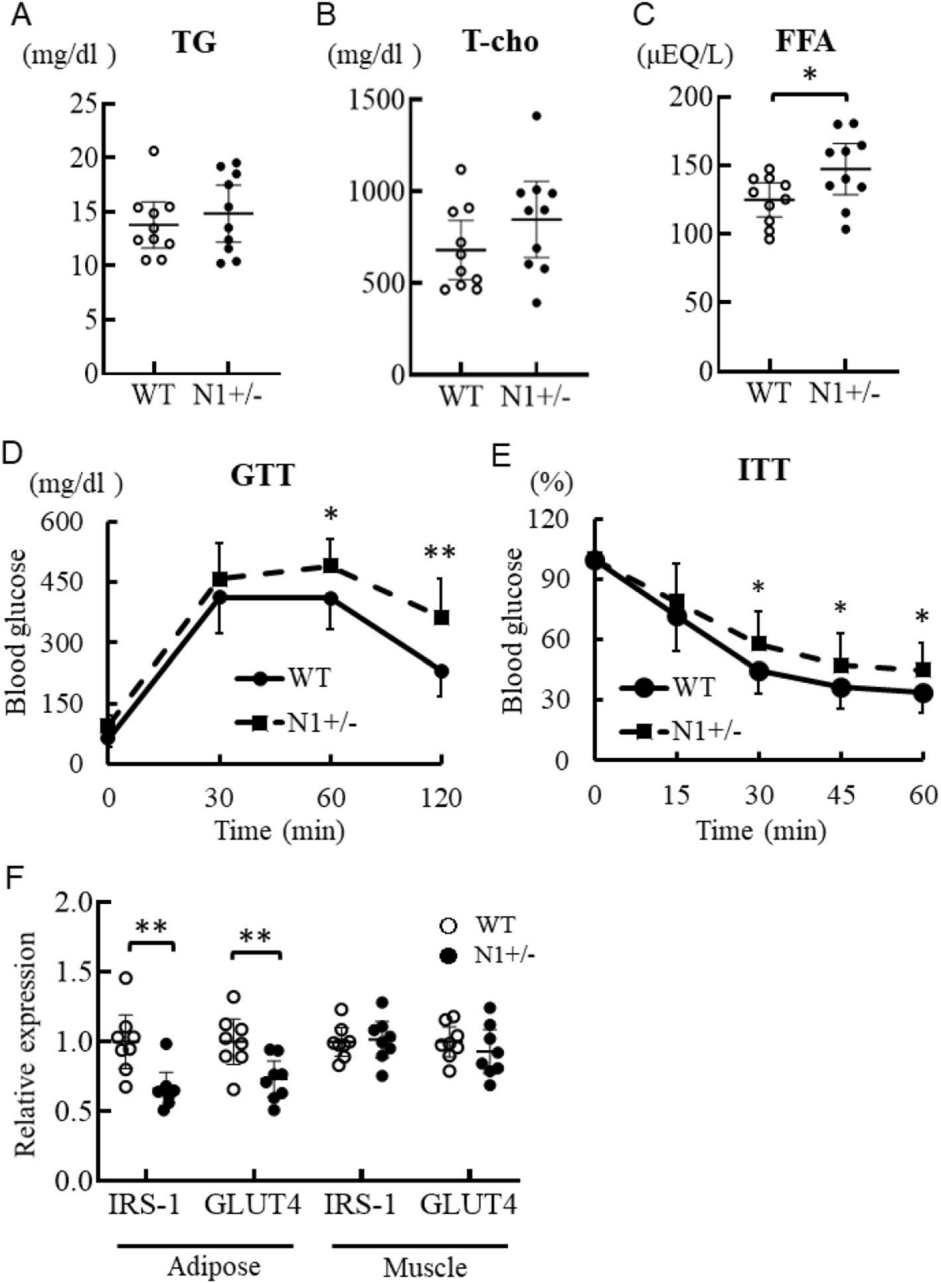
Notch1 haploinsufficiency alters systemic metabolism. Haploinsufficiency in Notch1 was associated with increased FFA concentration and worsening of glucose metabolism. Glucose and insulin tolerance in N1+/− mice deteriorated significantly after 12 weeks of HF/HS diet. In the N1+/− mice, the expression levels of IRS-1 and GLUT4 were lower in adipose tissue, but not in skeletal muscles. (**A**–**C**) Plasma fat and fatty acid composition in WT and N1+/− mice. Glucose tolerance test (**D**) and insulin tolerance test (**E**) in the WT and N1+/− mice. (**F**) Quantitative analysis of IRS-1 and GLUT4 mRNA expression levels in adipose tissue and skeletal muscle of WT and N1+/− mice. Data are mean ± SD. *P < 0.05 and **P < 0.01, compared with WT mice. n = 8–10 per group.

To investigate whether haploinsufficiency of Notch1 affects systemic metabolism, we assessed lipid composition, and glucose and insulin tolerance. Analysis of plasma lipid profile showed that Notch1 haploinsufficiency did not alter total cholesterol or triglyceride levels, but increased FFA concentration (Fig. 4A–C). Glucose and insulin tolerance were significantly worse in the 16-week-old HF/HS-fed N1+/− mice (Fig. 4D,E). We examined the mechanism underlying this effect on glucose tolerance and insulin tolerance in N1+/− mice by measuring the expression of IRS-1 and GLUT4 in adipose tissue and skeletal muscle. In the N1+/− mice, the expression of IRS-1 and GLUT4 was reduced in adipose tissue compared to the levels in WT mice, but not in skeletal muscle (Fig. 4F).

### In vitro experiments

#### Inhibition of Notch1 signaling promotes adipocyte differentiation and accumulation of lipid droplets

**Figure 5:**
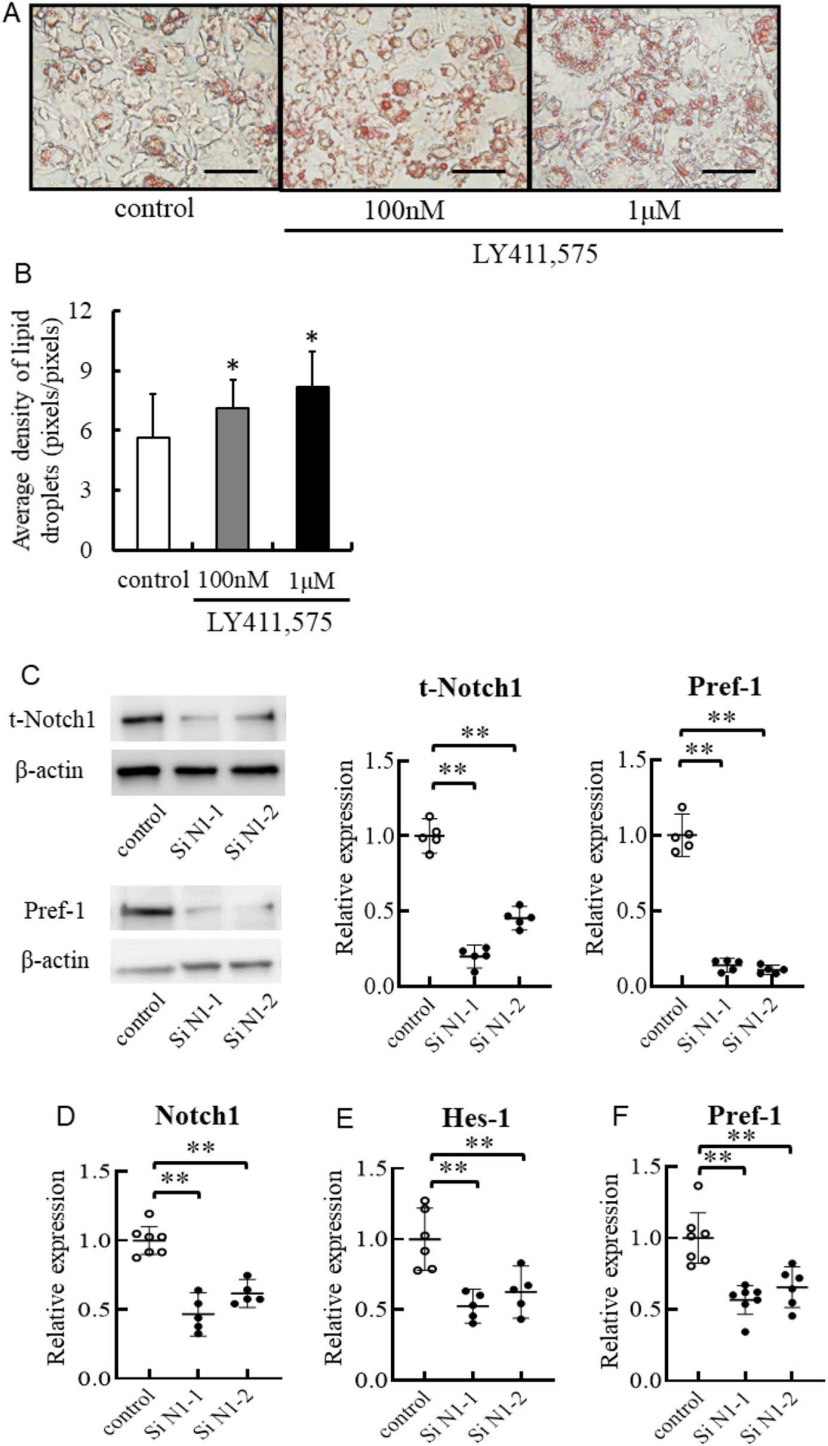

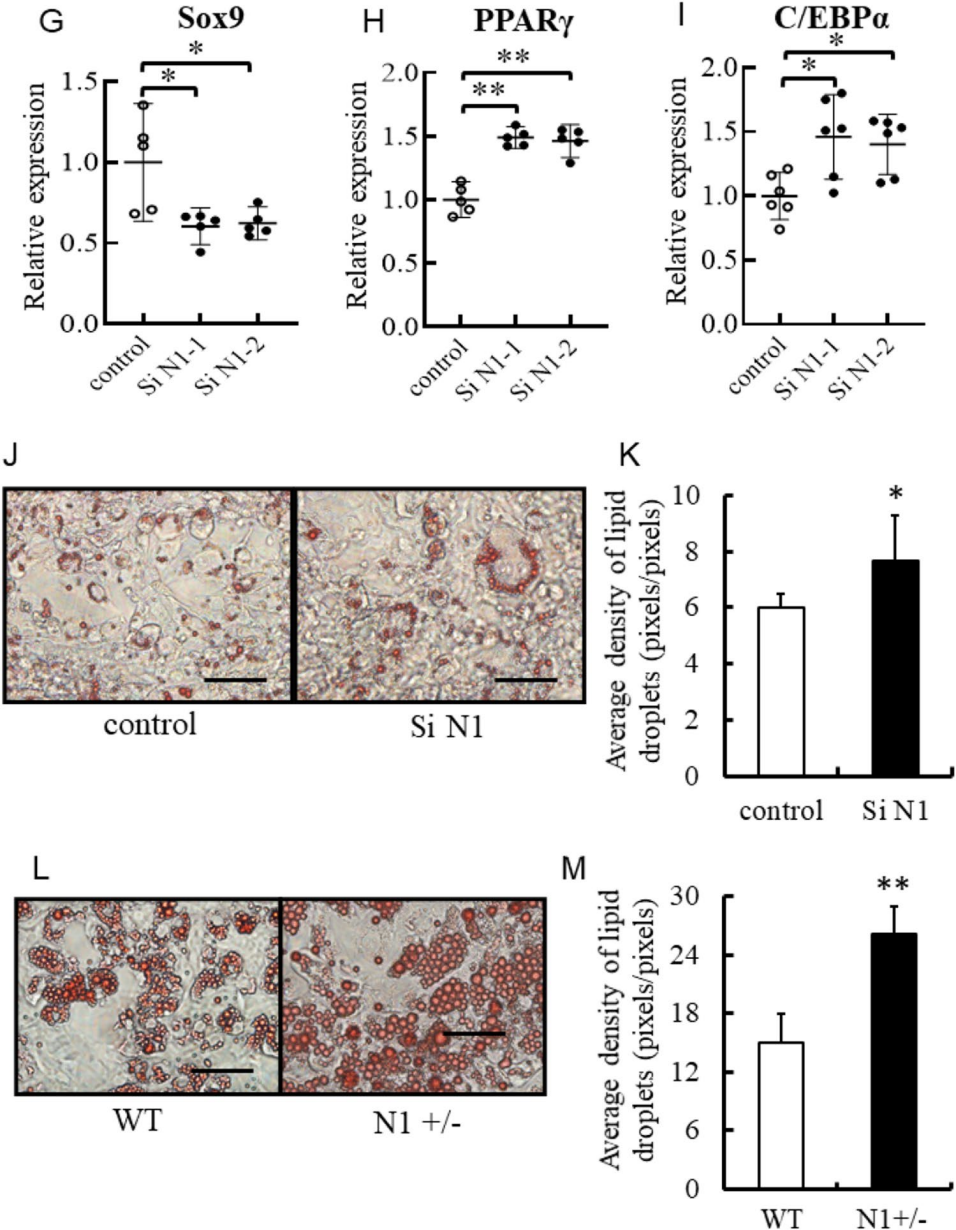
Notch1 signaling blockade promotes adipogenesis in vitro. Pharmacological Notch signal inhibition in 3T3-L1 cells, with γ-secretase inhibitors (LY411,575) increased adipocyte differentiation in a dose-dependent manner. Knockdown of Notch1 also increased adipocyte differentiation, followed by downregulation of Hes-1, Pref-1, and Sox9 and upregulation of PPARγ and C/EBPα. Note the presence of higher concentration of lipid droplets in adipose tissue-derived stem cells (ADSCs) of N1+/− mice relative to WT mice. (**A**) Representative images of oil red O staining. *Left*: control, *Middle*: 100 nM LY411,575, *Right*: 1 µM LY411,575. Scale bars = 20 μm. (**B**) Average density of lipid droplets. (**C**) Immunoblotting showing total-Notch1 and Pref-1 in 3T3-L1 cells. Representative western blot of fractionated samples. The cropped blots are used in the figure. The membranes were cut prior to exposure so that only the portion of gel containing the desired bands would be visualized. The samples of fractionation derive from the same experiment and the blots were processed in parallel. Full-length blots are shown in Supplementary information (Page 6). Quantification of western blot bands. Protein expression was normalized to β-actin. (**D**–**I**) Quantitative analysis of mRNA expression levels of Notch1 (**D**), Hes-1 (**E**), Pref-1 (**F**), Sox9 (**G**), PPARγ (**H**), and C/EBPα (**I**) in 3T3-L1 cells. (**J**) Representative images of oil red O staining. *Left*: control, Right: si-Notch1. Scale bars = 20 μm. (**K**) Average density of lipid droplets. (**L**) Representative images of oil red O staining. *Left*: control, *Right*: N1+/−. (**M**) Average density of lipid droplets in ADSCs. Scale bars = 20 μm Data are mean ± SD. *P < 0.05, compared with the control. n = 5–7 per group.

To determine whether Notch1 signaling blockade promotes adipogenesis, we used 3T3-L1 preadipocytes stained with Oil red O to assess the effects of pharmacological inhibition and knockdown of Notch1 before differentiation. Pharmacological Notch signal inhibition with LY411,575 decreased cleaved Notch1 and 3 and dose-dependently suppressed Hes-1 and Pref-1 (Supplemental Figure S2). Furthermore, Pharmacological Notch signal inhibition significantly increased adipocyte differentiation in a dose-dependent manner (Fig. 5A,B). Notch1 knockdown reduced Notch1 mRNA and protein expression (Fig. 5C). In parallel with the downregulation of Notch1 and the downstream transcriptional factor, Hes-1, the expression level of Sox9 was reduced, whereas those of PPARγ and C/EBPα were increased (Fig. 5D–I). Notably, Pref-1 protein also reduced accordant to reduced Notch1 signal (Fig. 5C). Finally, transfection of siRNA for Notch1 significantly increased adipocyte differentiation compared with nonsilencing control RNA (Fig. 5J,K). Furthermore, ADSCs obtained from 8-week-old N1+/− and WT mice were established and investigated for their adipogenic capacity. In the ADSCs of N1+/− , Notch1 protein expression was decreased without changes in Notch3 expression level (Supplemental Figure 3A). In the downstream of reduced Notch1 signal, Hes-1 and Pref-1 were reduced (Supplemental Figure 3B–D). Accumulation of lipid droplets was significantly more pronounced in ADSCs from N1+/− mice compared with those from WT mice (Fig. 5L,M).

## Discussion

Our study demonstrated that haploinsufficiency of Notch1 promotes fat accumulation and adipogenesis, and provides a mechanistic link between Notch signaling and development of insulin resistance (Figs. 1, 2 and 4). Adipose Notch1 in WT mice increased with fat accumulation, but the increment in N1+/− mice was blunted up to half of that in WT mice (Fig. 3). Our in vitro experiments using 3T3-L1 adipocytes showed increased expression of two of the main adipogenic factors (PPARγ and C/EBPα) in response to decreases in the levels of transcription factors downstream of Notch signaling and anti-adipogenesis (Hes-1, Pref-1, and Sox9), together with enhanced adipogenesis in adipose tissues with transformation of ADCSs obtained from N1+/− mice (Figs. 3 and 5). Pharmacological Notch signal inhibition and Notch1 knockdown by siRNA also promoted adipogenesis in 3T3-L1 preadipocytes (Fig. 5).

Our results showed significant increase in body weight and inguinal fat accumulation in N1+/− mice after 12 weeks of age, and that such increase was not related to any increase in food consumption (Fig. 1). Fat accumulation depends on both hypertrophy of preexisting adipocytes and hyperplasia due to formation of new adipocytes from precursor cells^14^. We examined markers of adipogenesis at both early stage in 8-week-old mice and also at later stage in 16-week-old mice. The results showed that increases in adipogenic cell clusters and proliferation markers were associated in N1+/− mice with enhanced adipogenesis (Fig. 2)^14^. Furthermore, the observed increase in crown-like structures in N1+/− was associated with maturation of adipocytes (Fig. 2)^14^. Interestingly, accumulation of adipocytes induced by enhanced adipogenesis and adipocyte hypertrophy was associated with the finding of abundant adipocytes of various sizes (Fig. 2). Despite the enhanced adipogenesis, accumulation of hypertrophic adipocyte and lipotoxicity caused by FFA release was associated with obesity-induced insulin resistance (Figs. 2 and 4)^16,18^. As FFA stimulates G-protein-coupled receptors such as GPR120 to promote adipogenesis^19^, FFA and Notch1 signal pathway would synergically promote adipose proliferation (Fig. 2F,G).

Similar to endothelial cells^2^, Notch1 expression was decreased in adipocytes by about 50% in N1+/− mice (Fig. 3B). This finding suggests that Notch1, rather than other homologs, plays a critical role in adipogenesis. Notch1 expression in adipose tissues reached a peak level at 8 weeks of age in WT mice, in parallel with fat accumulation, especially under HF/HS feeding condition. On the other hand, only a blunted increase in Notch1 was observed in 8-week-old N1+/− mice, which was only about half of that in WT mice (Fig. 3). Previous studies reported enhanced adipose tissue browning with improved energy homeostasis in adipose-specific Notch1 knockout mice, in which Notch1 expression in mature adipocytes was 85% lower than that of WT mice^20^. We did not find any evidence for browning of white adipose tissue in our N1+/− mice, as evident by analysis of various molecular markers and results of cold tolerance test, but showed phenotypes of obese and inadequate Notch1 signal suppression. The difference in the findings of the two studies could be related to the timing of activation and dosage effects on downstream transcription factors of Notch1 signaling^7,8^. In the previous study, chimeric mice of WT and N1−/− embryonic stem cells (ES cells) were generated to analyze whether N1−/− ES cells could differentiate into adipocytes in the adult body^21^. N1−/− ES cell as well as WT ES cells were detected as adipocytes in sebaceous gland^21^. Furthermore, Notch1 ± and Notch1−/− ES cells differentiated into adipocytes in in vitro embryoid body differentiation assay without statistical significance^21^. Thus, the Notch1 signal had been defined as an indispensable signaling pathway involved in the regulation of cell proliferation and differentiation of adipocytes^21^. In the recent studies, the results of Notch1 signal inhibition in vitro studies on adipocytes vary widely probably due to differences in the study protocols (e.g., pharmacological or genetic interference, and time course)^4^. This would suggest that the effect of downregulation of Notch1 expression in adipose tissue varies according to the timing and degree of suppression, and that there is not always linear correlation between dose of Notch1 allele and adipogenesis. The Notch1 signal pathway in T-cell acute lymphoblastic leukemia (T-ALL) has attracted attention as a therapeutic target to modulate cell signaling and metabolism^22^. Interestingly, T-ALL blasts with resistance to Notch1 inhibition revealed fainted Notch1 signaling and dis-regulated lipid metabolism pathway such as cholesterol homeostasis, adipogenesis and fatty acid metabolism^22^. This would suggest that Notch1 pathway inhibition also modulates lipid droplet formation in adipocytes via altered lipid metabolism.

Notch1 signaling is defined as a potent regulator of the adipogenesis process, but conflicting findings have been reported concerning the roles of Notch1 signaling in differentiation of pre-adipocytes^4^. Previous studies reported that overexpression of Notch ligand, Jagged, and Notch1 downstream transcription factor, Hes-1, inhibit adipogenesis in 3T3 L1 preadipocytes^23^. In contrast, pharmacological Notch signal inhibition decreased Hes-1 and increased Pref-1 in 3T3 L1 preadipocytes and promoted adipogenesis^24^. Thus, it seems that Pref-1 is an important target of Notch signaling for adipogenesis. Pref-1 is abundantly present in preadipocytes where it plays a role in negative regulation of adipogenic differentiation, and is not expressed in mature adipocytes^6^. Structurally, Pref-1 (also known as a delta-like 1 protein) is a transmembrane protein with a large extracellular part consisting of multimeric EGF-like repeats and a short juxtamembrane domain, a transmembrane helix and a short intracellular C-terminus^25^. The extracellular part is cleaved by TACE to generate a biologically active soluble form that interacts with fibronectin^25^. The fibronectin/soluble Pref-1 complex activates α5β1 integrin downstream signaling to upregulate SOX9 via the MEK/ERK pathway. SOX9 binds to the promoter regions of C/EBP β/δ and prevents the activation of C/EBPα and PPARγ to suppress adipocyte differentiation^6^. In the present study, haploinsufficiency of Notch1 in murine adipose tissues and ADSCs, and knockdown of Notch1 by siRNA and pharmacological Notch signal inhibition in 3T3 L1 cells also decreased Pref-1 and Sox9 in pre-adipocytes, with resultant increase in PPARγ and C/EBPα and enhanced adipogenesis. In the present study, the expression levels of anti-adipogenic molecules, such as Pref-1 and Sox9, increased in adipose tissues of obese WT mice downstream of Notch1 activation.

ADSCs reside within the stromal-vascular fraction of tissues, and are characterized as a heterogeneous cell population that express surface markers of mesenchymal stromal cells (CD34, CD73, CD44, CD90 and CD105) and preadipocytes (CD24 and CD29). In the present study, we found that haploinsufficiency of Notch1 enhanced adipogenesis in ADSCs derived from visceral fat^26^. Interestingly, Notch1 signaling is inherently activated in ADSCs derived from visceral fat, and pharmacological Notch signal inhibition promotes adipogenesis in ADSCs^27^. Generally, Notch signaling is a juxtacrine communication pathway between signal-sending cells expressing agonistic Notch ligands, and signal-receiving cells expressing Notch receptors to regulate stem cell maintenance in niche stromal cells^28^. This suggests the involvement of Notch1 signal activity in the stemness of ADSCs, and that modulation of Notch1 signaling in ADSCs is a potentially useful therapeutic target in obesity.

Notch1 signal has attracted attention in recent years as a novel regulator of metabolism^29^. Evidence suggests that Notch signaling accelerates gluconeogenesis and lipogenesis in the liver, which lead to hyperglycemia and fatty liver disease^30^. In the immune system, activation of Notch signaling promotes M1 macrophage polarization, producing a systemic low-grade inflammation state that exacerbates insulin resistance in peripheral tissues^29^. In the present study, N1+/− mice exhibited metabolic dysregulation with high FFA concentration, and altered glucose tolerance and insulin sensitivity in adipose tissue (Fig. 4). We focused on the activity of Notch signaling pathway in adipose tissue and skeletal muscle with the aim of determining the mechanism of systemic insulin resistance, since the adipose tissue accounts for about 15–20% of insulin-stimulated glucose uptake. We also focused on the skeletal muscle since it is responsible for about 80% of whole-body insulin-mediated glucose metabolism^31^. Notch1 haploinsufficiency in hepatocytes would contribute to insulin sensitivity^30^, but this would affect little on systemic insulin resistance. Our results showed downregulation of IRS-1 and GLUT4 limited to the adipose tissue of N1+/− mice, but not in skeletal muscles. To date, the direct mechanism(s) involved in Notch1 signal-related regulation of IRS-1 and GLUT4 expression remain unknown. At this stage, however, we know that Notch-1 signaling regulates cell survival through Hes-1-PTEN-AKT-mTOR signaling and Notch1 internal domain-mTORC2-AKT-mTOR signaling, which regulates the expression of these molecules^32^. Hypertrophic obesity is also associated with increased lipolysis, which increases the release of FFA by hypertrophic visceral adipose tissue and promotes insulin resistance^33^. Thus, haploinsufficiency of Notch1 in adipose tissue can result in dysregulation of glucose metabolism followed by Notch1-related and unrelated mechanisms.

In conclusion, we demonstrated in the present study that haploinsufficiency of Notch1 promotes adipose tissue accumulation and modulates adipogenic signaling, resulting in obesity-induced insulin resistance. These results suggest that Notch1 signaling could be involved in the development and management of obesity.

## Supplementary Information

Supplementary Information.

## References

[1] Hori K, Sen A, Artavanis-Tsakonas S (2013). Notch signaling at a glance. J. Cell Sci..

[2] Takeshita K (2007). Critical role of endothelial Notch1 signaling in postnatal angiogenesis. Circ. Res..

[3] Gridley T, Kajimura S (2014). Lightening up a notch: Notch regulation of energy metabolism. Nat. Med..

[4] Shan T, Liu J, Wu W, Xu Z, Wang Y (2017). Roles of Notch signaling in adipocyte progenitor cells and mature adipocytes. J. Cell. Physiol..

[5] Madsen MS, Siersbaek R, Boergesen M, Nielsen R, Mandrup S (2014). Peroxisome proliferator-activated receptor gamma and C/EBPalpha synergistically activate key metabolic adipocyte genes by assisted loading. Mol. Cell. Biol..

[6] da Silva C, Durandt C, Kallmeyer K, Ambele MA, Pepper MS (2020). The role of Pref-1 during adipogenic differentiation: An overview of suggested mechanisms. Int. J. Mol. Sci.

[7] Sueda R, Kageyama R (2020). Regulation of active and quiescent somatic stem cells by Notch signaling. Dev. Growth Differ..

[8] Theodoris CV (2015). Human disease modeling reveals integrated transcriptional and epigenetic mechanisms of NOTCH1 haploinsufficiency. Cell.

[9] Kikuchi R (2011). Pitavastatin-induced angiogenesis and arteriogenesis is mediated by Notch1 in a murine hindlimb ischemia model without induction of VEGF. Lab. Investig. J. Tech. Methods Pathol..

[10] Aoyama T (2009). gamma-Secretase inhibitor reduces diet-induced atherosclerosis in apolipoprotein E-deficient mice. Biochem. Biophys. Res. Commun..

[11] Yonezawa R (2012). Central versus peripheral impact of estradiol on the impaired glucose metabolism in ovariectomized mice on a high-fat diet. Am. J. Physiol. Endocrinol. Metab..

[12] Johnson EA, O'Callaghan JP, Miller DB (2004). Brain concentrations of d-MDMA are increased after stress. Psychopharmacology.

[13] Cinti S (2005). Adipocyte death defines macrophage localization and function in adipose tissue of obese mice and humans. J. Lipid Res..

[14] Nishimura S (2007). Adipogenesis in obesity requires close interplay between differentiating adipocytes, stromal cells, and blood vessels. Diabetes.

[15] Takeshita K (2002). Increased expression of plasminogen activator inhibitor-1 with fibrin deposition in a murine model of aging, "Klotho" mouse. Semin. Thromb. Hemost..

[16] Uchida Y (2012). Stress augments insulin resistance and prothrombotic state: Role of visceral adipose-derived monocyte chemoattractant protein-1. Diabetes.

[17] Lei Y (2017). Increased dipeptidyl peptidase-4 accelerates diet-related vascular aging and atherosclerosis in ApoE-deficient mice under chronic stress. Int. J. Cardiol..

[18] Abdul-Ghani MA (2008). Deleterious action of FA metabolites on ATP synthesis: Possible link between lipotoxicity, mitochondrial dysfunction, and insulin resistance. Am. J. Physiol. Endocrinol. Metab..

[19] Ichimura A, Hara T, Hirasawa A (2014). Regulation of energy homeostasis via GPR120. Front. Endocrinol..

[20] Bi P (2014). Inhibition of Notch signaling promotes browning of white adipose tissue and ameliorates obesity. Nat. Med..

[21] Nichols AM (2004). Notch pathway is dispensable for adipocyte specification. Genesis.

[22] Agnusdei V (2020). Dissecting molecular mechanisms of resistance to NOTCH1-targeted therapy in T-cell acute lymphoblastic leukemia xenografts. Haematologica.

[23] Ross DA, Rao PK, Kadesch T (2004). Dual roles for the Notch target gene Hes-1 in the differentiation of 3T3-L1 preadipocytes. Mol. Cell. Biol..

[24] Huang Y (2010). gamma-secretase inhibitor induces adipogenesis of adipose-derived stem cells by regulation of Notch and PPAR-gamma. Cell Prolif..

[25] Sul HS (2009). Minireview: Pref-1: Role in adipogenesis and mesenchymal cell fate. Mol. Endocrinol..

[26] Si Z (2019). Adipose-derived stem cells: Sources, potency, and implications for regenerative therapies. Biomed. Pharmacother..

[27] Ferrer-Lorente R, Bejar MT, Badimon L (2014). Notch signaling pathway activation in normal and hyperglycemic rats differs in the stem cells of visceral and subcutaneous adipose tissue. Stem Cells Dev..

[28] Vanderbeck AN, Maillard I (2019). Notch in the niche: New insights into the role of Notch signaling in the bone marrow. Haematologica.

[29] Bi P, Kuang S (2015). Notch signaling as a novel regulator of metabolism. Trends Endocrinol. Metab..

[30] Pajvani UB (2013). Inhibition of Notch uncouples Akt activation from hepatic lipid accumulation by decreasing mTorc1 stability. Nat. Med..

[31] Shulman GI (1990). Quantitation of muscle glycogen synthesis in normal subjects and subjects with non-insulin-dependent diabetes by 13C nuclear magnetic resonance spectroscopy. N. Engl. J. Med..

[32] Zeng C, Xing R, Liu J, Xing F (2016). Role of CSL-dependent and independent Notch signaling pathways in cell apoptosis. Apoptosis Int. J. Program. Cell Death.

[33] Hammarstedt A, Gogg S, Hedjazifar S, Nerstedt A, Smith U (2018). Impaired adipogenesis and dysfunctional adipose tissue in human hypertrophic obesity. Physiol. Rev..

