# Supplementary material for: Notch1 haploinsufficiency in mice accelerates adipogenesis
Source: Sci Rep. 2021 Aug 18;11:16761. doi: 10.1038/s41598-021-96017-z (PMC8373919; doi:10.1038/s41598-021-96017-z)

**Notch1 Haploinsufficiency in Mice Accelerates Adipogenesis**

Kazutoshi Yamaguchi ^1^, Motoharu Hayashi ^1^, Yasuhiro Uchida ^1^, Xian Wu Cheng ^2, 3^, Takayuki Nakayama ^4^, Tadashi Matsushita ^5, 6^, Toyoaki Murohara ^1^, Kyosuke Takeshita ^1, 5, 7^*

^1^Department of Cardiology, Nagoya University Graduate School of Medicine, Nagoya, Japan.

^2^Department of Cardiology/Hypertension and Heart Center, Yanbian University Hospital, Yanji, Jilin, China.

^3^Department of Community Health and Geriatrics, Nagoya University Graduate School of Medicine, Nagoya, Japan.

^4^Department of Blood Transfusion, Aichi Medical University Hospital, Nagakute, Japan.

^5^Department of Clinical Laboratory, Nagoya University Hospital, Nagoya, Japan.

^6^Department of Blood Transfusion, Nagoya University Hospital, Nagoya, Japan.

^7^Department of Clinical Laboratory, Saitama Medical Centre, Saitama Medical University, Kawagoe, Japan.

*****Corresponding author: Prof. Kyosuke Takeshita, MD, PhD, FAHA. Department of Clinical Laboratory, Saitama Medical Centre, Saitama Medical University, 1981 Kamoda Kawagoe, Saitama, Japan. Tel: +81 52 744 2147; Fax: +81 52 744 2138. E-mail: kyousuke@saitama-med.ac.jp.

**
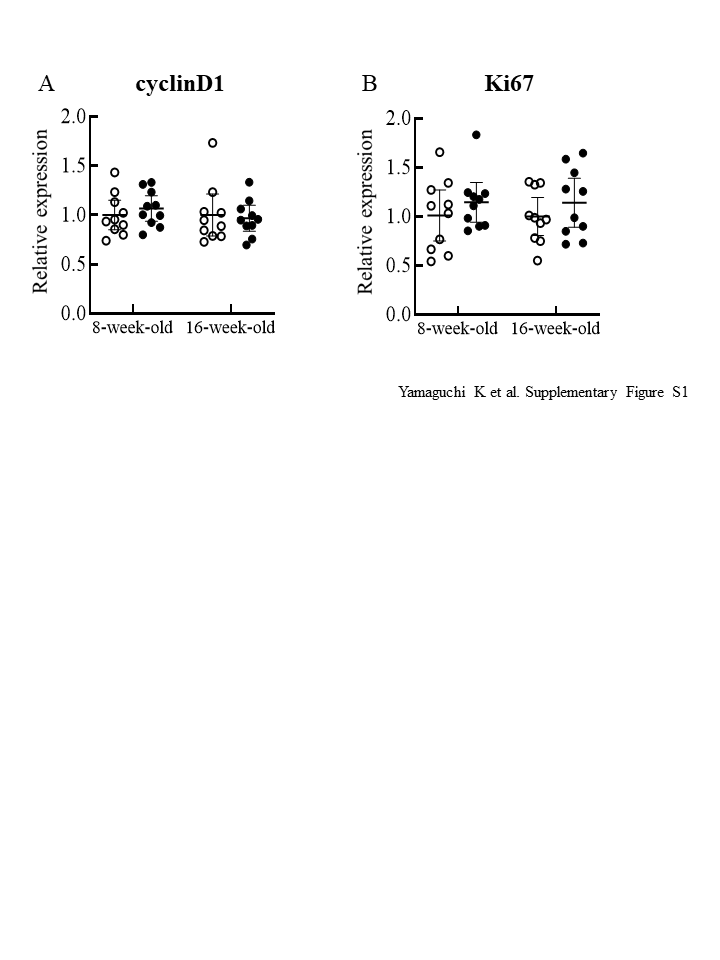
**

**Supplementary Figure S1**

Quantitative analysis of cyclin D1 (A) and Ki67 (B) mRNA in adipose tissue of WT and N1+/- mice, fed with normal diet. Data are mean±SD. n=10 per group.

**
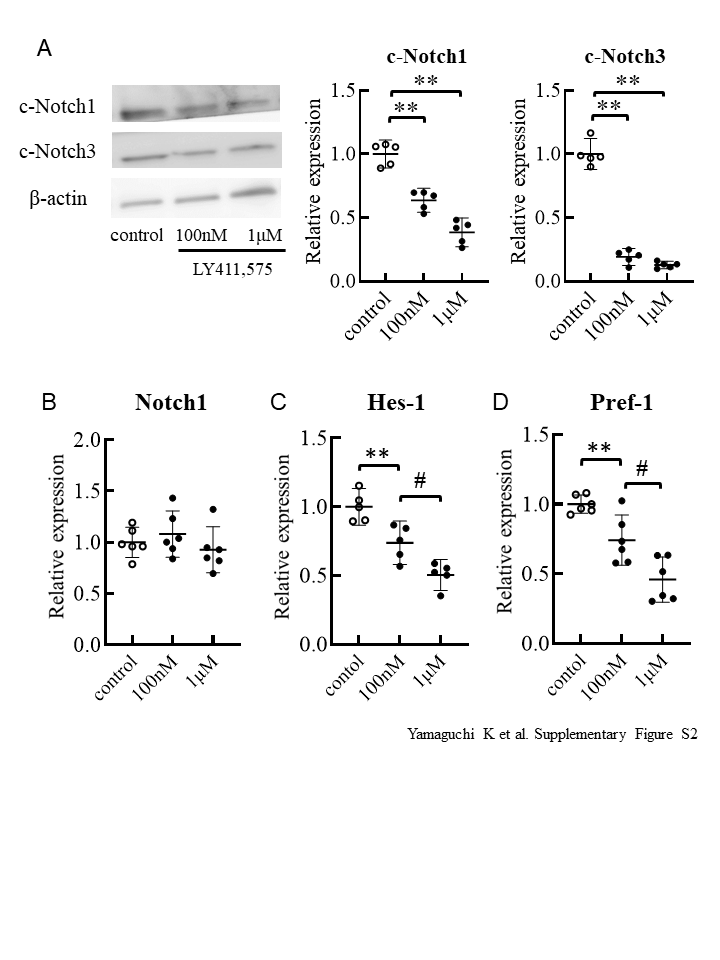
**

**Supplementary Figure S2**

(A) Immunoblotting showing cleaved-Notch1 and cleaved-Notch3 in 3T3-L1 cells, with γ-secretase inhibitors (LY411,575). Representative western blot of fractionated samples. The cropped blots are used in the figure. The membranes were cut prior to exposure so that only the portion of gel containing the desired bands would be visualized. The samples of fractionation derive from the same experiment and the blots were processed in parallel. Full-length blots are shown in Supplementary information (Page 7). Quantification of western blot bands. Protein expression was normalized to β-actin. (B-D) Quantitative analysis of mRNA expression levels of Notch1 (B), Hes-1 (C), and Pref-1 (D) in 3T3-L1 cells with γ-secretase inhibitors (LY411,575). Data are mean±SD. **P<0.01 compared with control, #p<0.05, compared with 100nM group. n=5 or 6 per group.


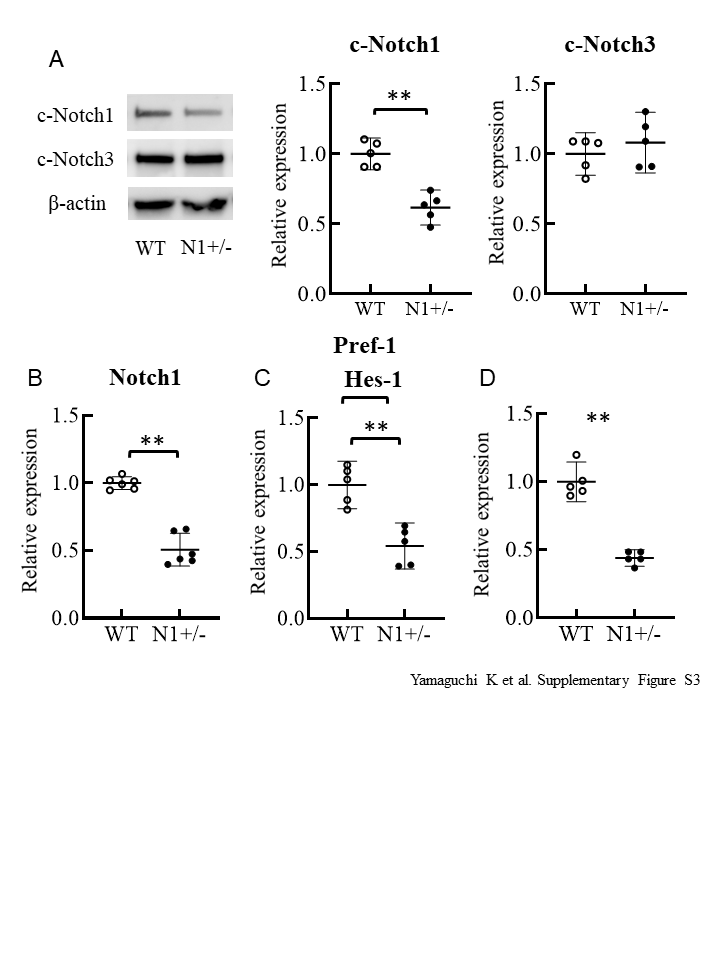


**Supplementary Figure S3**

(A) Immunoblotting showing cleaved-Notch1 and cleaved-Notch3 in adipose tissue-derived stem cells (ADSCs). Representative western blot of fractionated samples. The cropped blots are used in the figure. The membranes were cut prior to exposure so that only the portion of gel containing the desired bands would be visualized. The samples of fractionation derive from the same experiment and the blots were processed in parallel. Full-length blots are shown in Supplementary information (Page 7). Quantification of western blot bands. Protein expression was normalized to β-actin. (B-D) Quantitative analysis of mRNA expression levels of Notch1 (B), Hes-1 (C), and Pref-1 (D) in ADSCs of N1+/- and WT mice. Data are mean±SD. **P<0.01, compared with WT mice. n=5 or 6 per group.

Original blots


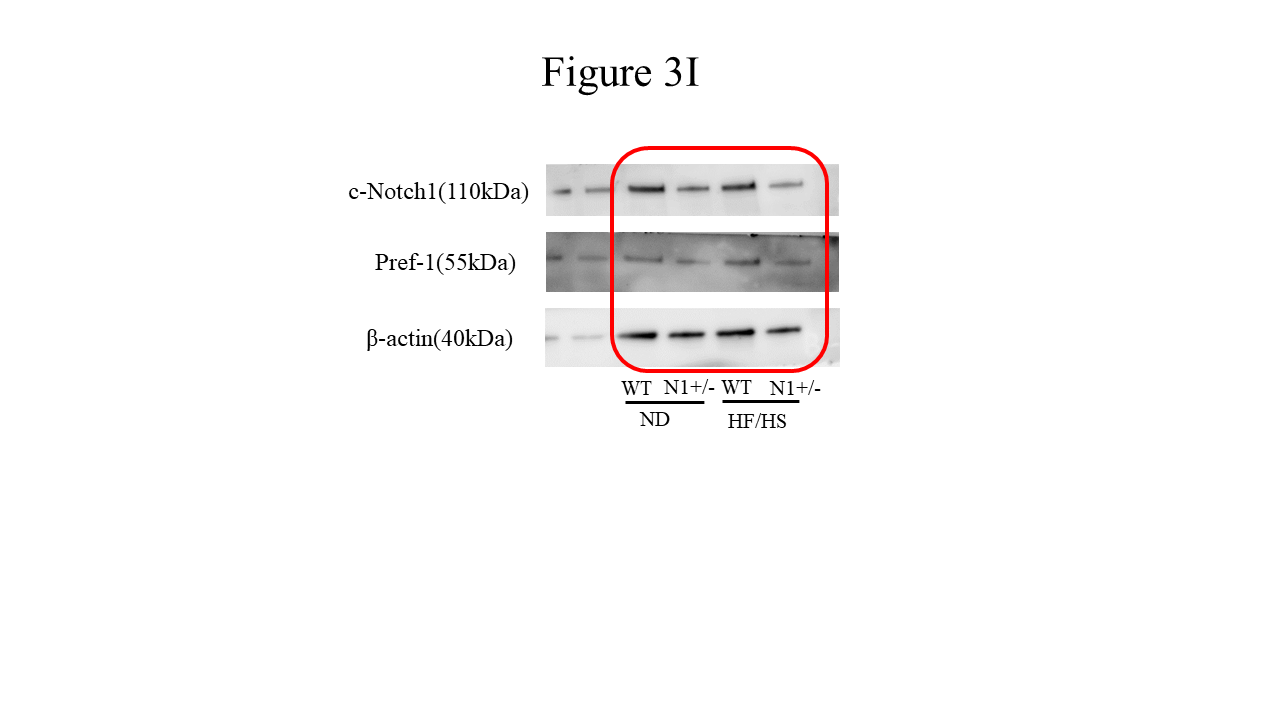

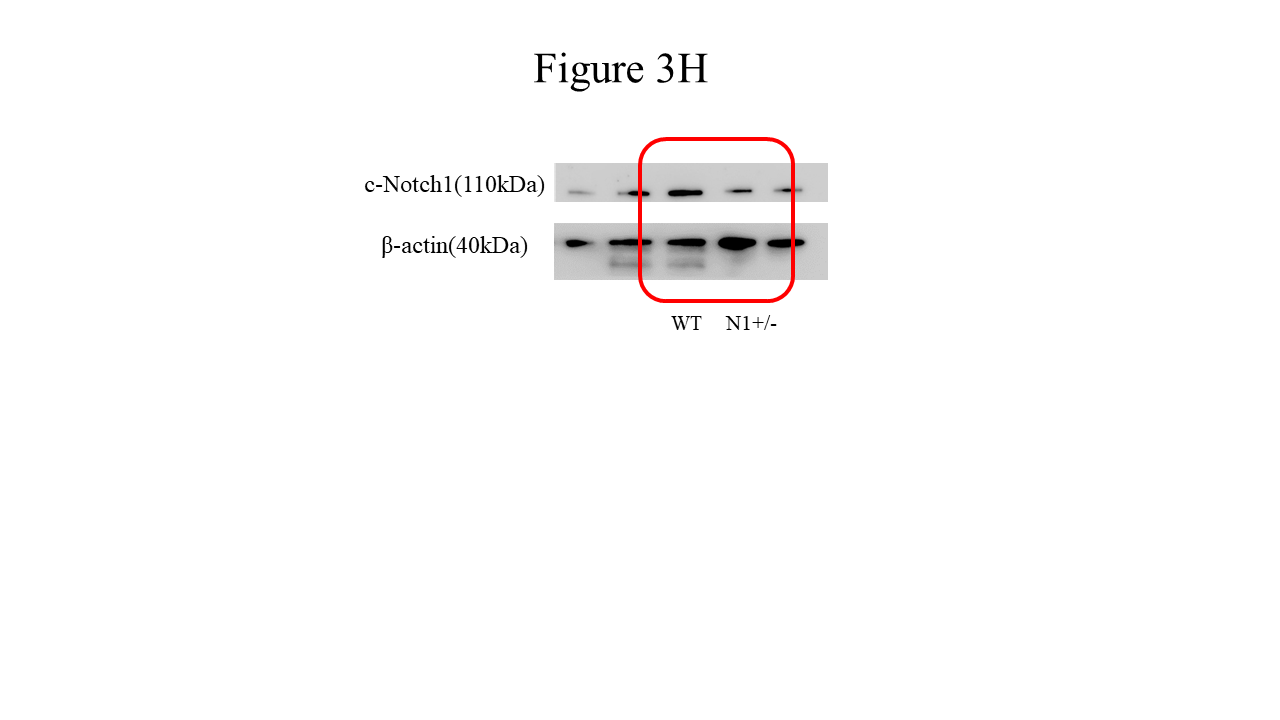

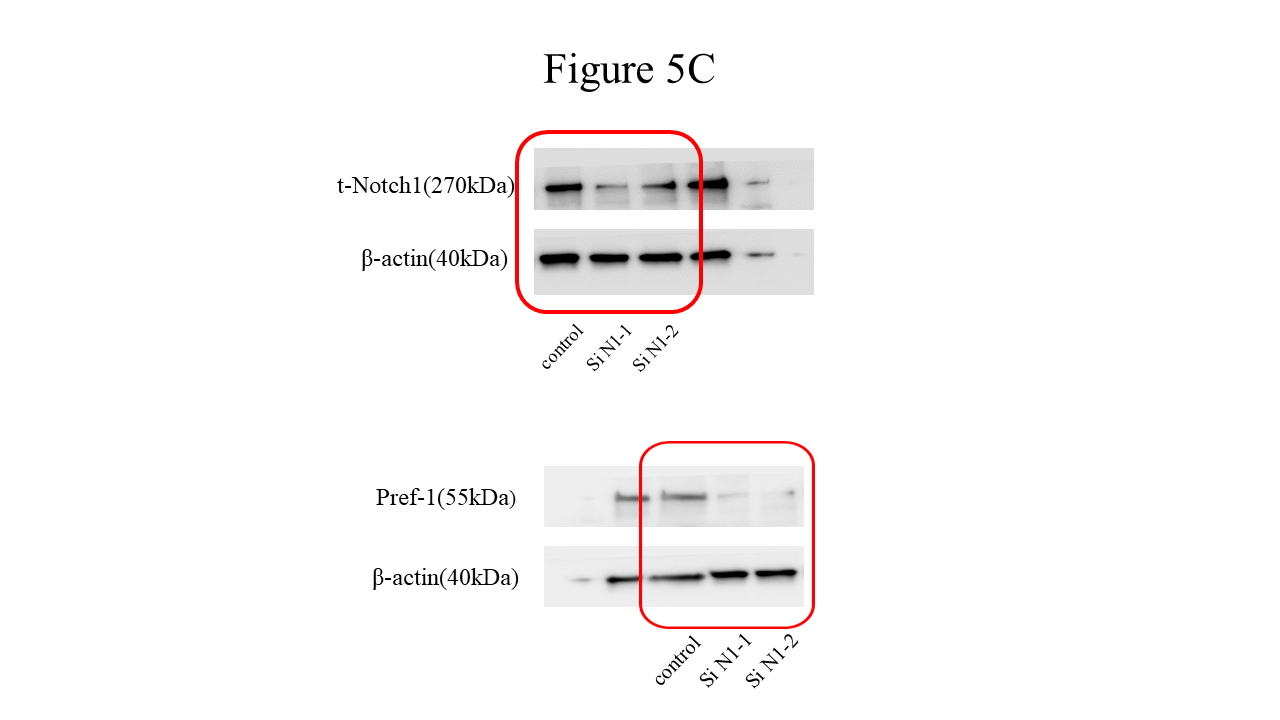

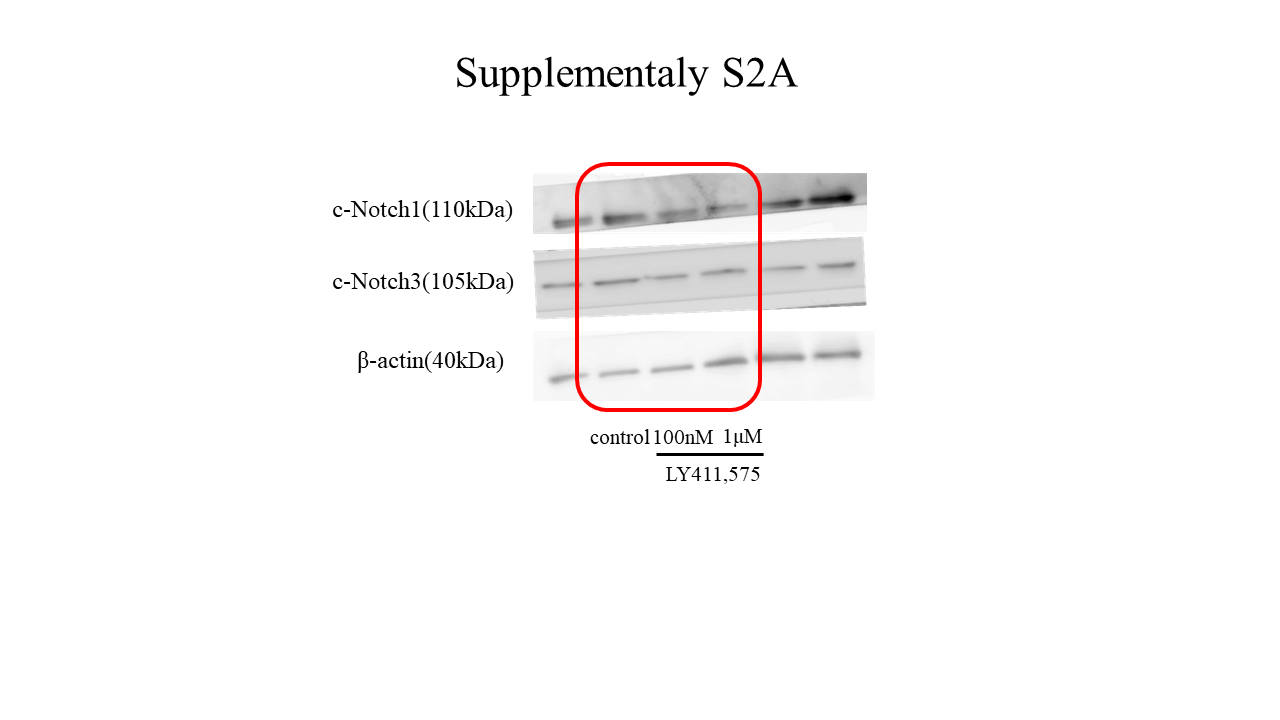

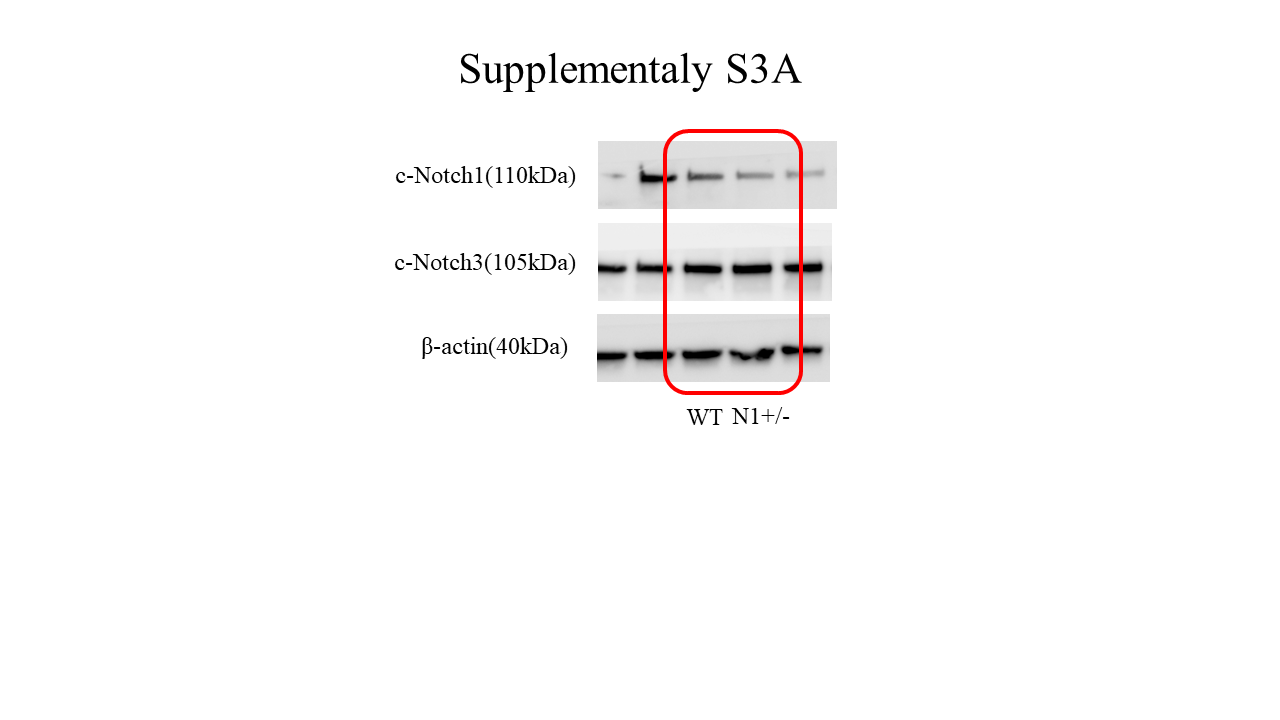

Supplement: Supplementary file 1 — Supplementary Information. [file 41598_2021_96017_MOESM1_ESM.docx]
